# STAT6 and STAT1 Pathway Activation in Circulating Lymphocytes and Monocytes as Predictor of Treatment Response in Rheumatoid Arthritis

**DOI:** 10.1371/journal.pone.0167975

**Published:** 2016-12-12

**Authors:** Krista Kuuliala, Antti Kuuliala, Riitta Koivuniemi, Hannu Kautiainen, Heikki Repo, Marjatta Leirisalo-Repo

**Affiliations:** 1 Bacteriology and immunology, Helsinki University Hospital and University of Helsinki, Helsinki, Finland; 2 Rheumatology, Helsinki University Hospital and University of Helsinki, Helsinki, Finland; 3 Primary Health Care, Helsinki University Hospital and University of Helsinki, Helsinki, Finland; 4 General Practice, Helsinki University Hospital and University of Helsinki, Helsinki, Finland; 5 Unit of Primary Health Care, Kuopio University Hospital, Kuopio, Finland; Ludwig-Maximilians-Universitat Munchen, GERMANY

## Abstract

**Objective:**

To find novel predictors of treatment response to disease-modifying antirheumatic drugs (DMARDs), we studied activation of STAT (signal transducers and activators of transcription) 6 and 1 in circulating leukocytes of patients with rheumatoid arthritis (RA).

**Methods:**

19 patients with untreated recent-onset RA, 16 patients with chronic RA irresponsive to synthetic DMARDs and 37 healthy volunteers provided blood samples for whole blood flow cytometric determination of intracellular STAT6 and STAT1 phosphorylation, expressed as relative fluorescence units, in response to IL-4 and IFN-γ, respectively. Phosphorylation was restudied and treatment response (according to European League Against Rheumatism) determined after 1-year treatment with synthetic DMARDs in recent-onset RA and with biological DMARD in synthetic DMARD-irresponsive RA. Estimation-based exact logistic regression was used to investigate relation of baseline variables to treatment response. 95% confidence intervals of means were estimated by bias-corrected bootstrapping and the significance between baseline and follow-up values was calculated by permutation test.

**Results:**

At baseline, levels of phosphorylated STAT6 (pSTAT6) induced by IL-4 in monocytes were higher in those who achieved good treatment response to synthetic DMARDs than in those who did not among patients with untreated RA (OR 2.74, 95% CI 1.05 to 9.47), and IFN-γ -stimulated lymphocyte pSTAT1 levels were higher in those who achieved good treatment response to a biological drug than in those who did not among patients with chronic RA (OR 3.91, 95% CI 1.12 to 20.68). During follow-up, in recent-onset RA patients with good treatment response to synthetic DMARDS, the lymphocyte pSTAT6 levels decreased (p = 0.011), and, consequently, the ratio of pSTAT1/pSTAT6 in lymphocytes increased (p = 0.042).

**Conclusion:**

Cytokine-stimulated STAT6 and STAT1 phosphorylation in circulating leukocytes was associated with treatment response to DMARDs in this pilot study. The result, if confirmed in larger studies, may aid in developing personalized medicine in RA.

## Introduction

Rheumatoid arthritis (RA) is a disease of autoimmune origin characterized by synovitis, autoantibody production, cartilage and bone destruction, and systemic inflammation. RA is predisposed by both genetic and environmental triggers, and complex adaptive and innate immune mechanisms contribute to the disease course. [1] The treatment of patients with newly diagnosed RA is usually started with traditional disease-modifying antirheumatic drugs (DMARDs), and intensified, if necessary, by biological drugs, most commonly inhibitors of the proinflammatory cytokines such as tumor necrosis factor (TNF) [2].

In order to maintain RA patients’ work capacity optimally, remission should be achieved rapidly [3,4]. However, at present there are virtually no reliable biomarkers to predict treatment response to the chosen treatment in RA. Several studies have been performed to examine the usefulness of clinical and laboratory variables, autoantibodies, cytokines and genetic factors as predictors of treatment response to methotrexate and other types of DMARDs as well as to anti-TNF agents and other biological drugs [5,6]. The most studied treatment response marker candidates may be plasma levels of inflammatory cytokines and other soluble mediators. However, the results concerning the treatment response marker ability of these candidates can be contradicting [5,7], or they may not provide additional predictive value to the levels of inflammatory activity markers that are already in clinical use (primarily C-reactive protein and erythrocyte sedimentation rate) [8–10]. Among demographics and clinical data, the treatment strategy seems to be the strongest predictor [11]. Rheumatoid factor and anticitrullinated protein antibodies may be used in clinical practice, as their presence has been reported to associate with good treatment response to biologicals [12–14], but not uniformly, either [15,16].

Immune cell profiling is a novel approach to find predictive markers in RA, including studies on cell surface marker determination [17–19], while studies on potential markers belonging to intracellular signaling in immune cells are rare so far. Such markers could be, for example, STAT (signal transduction and activator of transcription) family members, which are involved in leukocyte signaling in response to various cytokines and growth factors, become activated mainly by phosphorylation, and play important roles in immune responses [20]. STAT1 and STAT6, for example, are tempting targets for marker research on RA for several reasons. First, their expression is upregulated in synovial lymphocytes, macrophages and fibroblasts in inflammatory arthritis and diminishes along with successful response to DMARDs [21–23]. However, these STATs have quite divergent effects. STAT1 elicits the Th1 type of immune responses, interacts with Th17 type response development, activates inflammation, but also exerts homeostatic functions and attenuates tissue destruction [24]. STAT6 promotes expression of several Th2-specific transcription factors and subsequent production of Th2 cytokines, humoral immunity and regulatory T cell response [25,26]. Thus, the roles of STAT1 and STAT6 in the continuum of the inflammatory process in the joints apparently differ from each other, and remain unresolved yet. Second, despite the obvious importance of STAT1 and STAT6 on the immune response, their activation in circulating immune cells is not known, especially in relation to each other. Third, while the effects of both the principal STAT1-activating cytokine IFN-γ and STAT6-activating IL-4 seem either advantageous or disadvantageous depending on the phase of the arthritic disease or the animal model used [24,27,28], activation of STAT1 and STAT6 in response to these cytokines with respect to the RA progression and treatment response remain largely unknown.

To the present study we recruited patients with recent-onset RA who had not started DMARD therapy, another group of patients with chronic DMARD-irresponsive RA who started biological therapy, and a reference group of healthy subjects, and determined STAT1 and STAT6 phosphorylation in circulating leukocyte subsets in response to cytokine stimulation (IFN-γ and IL-4, respectively) using whole blood flow cytometry. The patients were examined at baseline and after one year of treatment and the data were analyzed in relation to the activity and outcome of RA. To our knowledge, this is the first study to explore intracellular signaling of both STAT1 and STAT6 pathways in circulating leukocyte subtypes with regard to treatment response in RA.

## Materials and Methods

### Subjects

The study comprised two patient groups and a healthy reference group. Nineteen patients who had been newly diagnosed with RA and not received DMARDs or oral corticosteroids prior to blood sampling were the recent-onset RA group. Sixteen patients with persisting disease activity despite treatment with several synthetic DMARDs were the chronic RA group. Their blood samples were obtained before initiating biological DMARD therapy. The patients were recruited at the Division of Rheumatology, Helsinki University Central Hospital, from July 2010 to March 2012. An additional group consisting of 37 healthy subjects was recruited among laboratory and hospital personnel who did not have autoimmune diseases or immunosuppressive medication. Their samples were used as references to ensure the comparability of the activation results during the time span of the study.

All patients fulfilled the ACR/EULAR 2010 classification criteria for RA [29]. The study protocol was approved by the Ethical Review Board of the Joint Authority for the Hospital District of Helsinki and Uusimaa. An informed written consent was obtained from each subject.

### Clinical evaluation

A comprehensive clinical and laboratory evaluation was undertaken at entry concomitant to blood sampling, and after follow-up time (median 10 months, range 5 to 24 months) to assess outcome. 66/68 joints were evaluated for swelling and pain, patient’s global assessment of disease activity was recorded on a 100 mm visual analogue scale, and laboratory measurements including erythrocyte sedimentation rate (ESR) and plasma C-reactive protein (CRP) level were logged. Disease activity score using 28 joints with ESR (DAS28) was calculated [30].

### Blood samples and leukocyte stimulation

A 4-ml blood sample was taken, at baseline and at follow-up, by venipuncture from the antecubital vein into a Falcon polypropylene tube (Becton Dickinson, Lincoln Park, NJ) supplemented with 400 μl of pyrogen-free acid citrate dextrose solution A (ACD-A, Baxter Health Care Ltd, Norfolk, UK). Aliquoting and stimulations were performed within 3 hours of blood sampling.

100-μl aliquots of blood were put into polystyrene tubes (BD) and stimulated either with human recombinant IL-4 (R&D Systems, Minneapolis, MN) at final concentration of 10 ng/ml for 10 min or with hrIFN-γ (R&D Systems) at final concentration of 100 ng/ml for 5 min at +37°C, or incubated without cytokine stimulation at +37°C. After aliquoting, the tubes were also supplemented with the monocyte surface marker antibody anti-CD14-FITC (mouse anti-human IgG2b, κ, clone MφP9) (5 μl) (Becton Dickinson Biosciences, San Jose, CA).

### Whole blood flow cytometric protocol

Blood samples were prepared using a protocol and reagents by Becton Dickinson [31]. Optimal amounts of the antibodies and their compatibility with the permeabilization procedure, as well as the cytokine stimulation conditions described above, were chosen based on preliminary experiments. Unstimulated samples were used as controls because they provide the best means to distinguish positive from negative events [32,33].

Following the initial incubations, leukocytes were fixed and erythrocytes lysed by adding 1X Lyse/Fix Buffer. After pelleting, leukocytes were washed with Stain Buffer and permeabilized by Perm Buffer III at -20°C for 30 min. Cells were pelleted and washed with Stain Buffer, after which the tubes were supplemented with the T cell marker antibody anti-CD3-PerCP (mouse anti-human IgG1, κ, clone SK7) (9 μl), and the IL-4 -stimulated tube and its unstimulated control tube with anti-STAT6 (pTyr641) -Alexa Fluor 647 antibody (mouse anti-human IgG2a, clone 18/P-Stat6) (5 μl), and the IFN-γ -stimulated tube and its unstimulated control tube with anti-STAT1 (pTyr701) -Alexa Fluor 647 antibody (mouse anti-human IgG2a, clone 4a) (5 μl), in 100 μl of Stain Buffer. Following incubation at room temperature protected from light for 20 min, cells were washed in Stain Buffer and resuspended in 300 μl of Stain Buffer. The samples were kept on ice for a maximum of 4 hours until flow cytometric acquisition.

**Fig 1:**
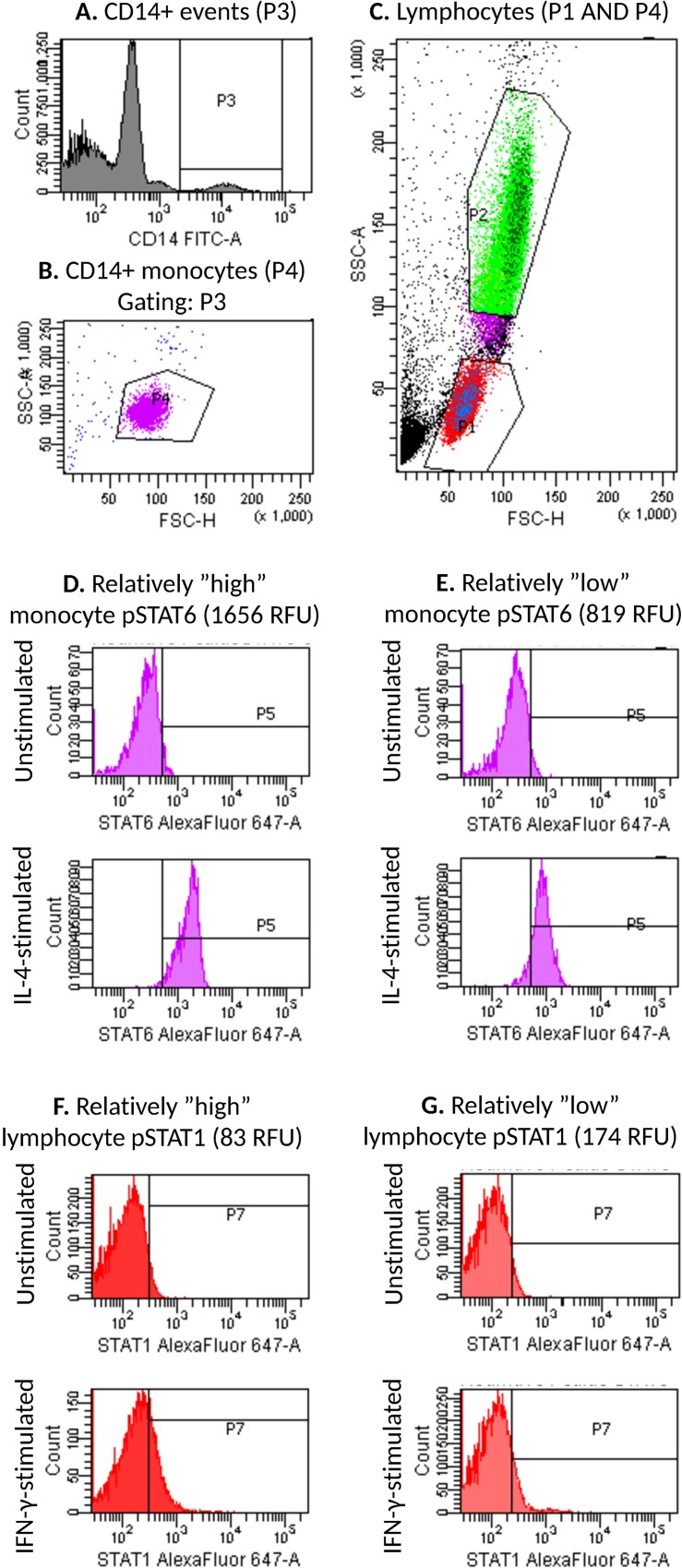
Flow cytometry gating strategy. A) Gate P3 was set to comprise all events with high CD14-FITC-fluorescence. B) Among events in P3, monocytes were included in gate P4 based on light scattering characteristics (FSC and SSC). C) Gate P1 was set to comprise lymphocytes based on light scattering characteristics, and for analysis all events in P1 but not in P4 were considered lymphocytes. Gate P2 was set to comprise neutrophils. pSTAT6-Alexa Fluor 647 and pSTAT1-Alexa Fluor 647 fluorescence intensity histograms were created for lymphocytes and monocytes in respective tubes. Representative histograms are shown for comparatively high (D) and low (E) monocyte pSTAT6 activation, and comparatively high (F) and low (G) lymphocyte pSTAT1 activation. Gate P5 (D, E) or P7 (F, G) is set to comprise 2–4% of events in the unstimulated sample and copied to the corresponding stimulated sample, *i*.*e*. the unstimulated samples serve as controls for the stimulated samples.

Flow cytometric data were acquired on FACSCantoII flow cytometer and analyzed with FACSDiva software (BD), as described previously [34]. Monocytes were identified by their CD14-positivity and light scattering characteristics (Fig 1A and 1B). Lymphocytes were identified by their light scattering characteristics (Fig 1C). pSTAT1 and pSTAT6 fluorescence intensity histograms were created for both stimulated and unstimulated lymphocytes and monocytes (for examples, see Fig 1D–1G). The intensities were expressed as relative fluorescence units (RFU).

### Statistical analysis

The data are presented as means with standard deviations (SD), medians with interquartile range (IQR), or counts with percentages. Correlations were estimated by Spearman’s correlation coefficient method. Estimation-based exact logistic regression was used to investigate relation of baseline variables to treatment response. In all analyses, patients with good EULAR response were categorized as good responders and patients with moderate or no response as non-responders. The 95% confidence intervals (CI) of means were estimated by bias-corrected bootstrapping (5000 replications) and the significance of difference between baseline and follow-up values was calculated by permutation test for paired replicates. Stata 13.1 (StataCorp LP, College Station, TX, USA) statistical package was used for the analyses.

## Results

### Subjects and treatment

A total of 19 patients had recent-onset RA with a median duration of symptoms of 12 months (interquartile range 4 to 24 months) and had not previously received any synthetic DMARDs or oral glucocorticoids (Table 1). Another 16 patients had chronic RA with median disease duration of 12.5 years (interquartile range 7 to 15.5 years) and were included in the study prior to initiating their first biological DMARD (Table 1).

**Table 1:** Characteristics of the subjects at baseline.

Variables	Recent-onset RA (N = 19)	Chronic RA (N = 16)	Healthy subjects (N = 37)
*Demographics*			
Women, n (%)	16 (84)	13 (81)	24 (65%)
Age (years), mean (SD)	46 (15)	48 (14)	37 (15)
Rheumatoid factor positive, n (%)	15 (79)	13 (81)	
ACPA positive, n (%)	14 (74)	12 (80)	
*Measures of disease activity*, *mean (SD)*			
DAS28	3.79 (1.55)	4.79 (1.32)	
Erythrocyte sedimentation rate (mm/h)	24 (23)	24 (15)	
Plasma C-reactive protein (mg/l)	18 (26)	11 (9)	
Number of swollen joints (0–66)	7 (5)	11 (7)	
Number of tender joints (0–68)	7 (5)	12 (9)	
Patient’s global assessment (VAS 0–100 mm)	45 (24)	51 (23)	
*pSTAT6*, *RFU*, *mean (SD)*			
IL-4 –stimulated monocytes	1240 (339)	1094 (287)	1054 (266)
Unstimulated monocytes	247 (31)	235 (65)	233 (38)
IL-4 –stimulated lymphocytes	382 (108)	348 (103)	312 (70)
Unstimulated lymphocytes	95 (12)	92 (17)	89 (14)
*pSTAT1*, *RFU*, *mean (SD)*			
IFN-γ –stimulated monocytes	1967 (661)	1616 (537)	2087 (869)
Unstimulated monocytes	284 (39)	293 (48)	271 (33)
IFN-γ –stimulated lymphocytes	146 (20)	141 (24)	138 (21)
Unstimulated lymphocytes	109 (13)	199 (27)	100 (16)
*pSTAT1/pSTAT6 ratio*, *mean (SD)*			
Monocytes	1.63 (0.50)	1.55 (0.56)	2.02 (0.74)
Lymphocytes	0.41 (0.11)	0.43 (0.12)	0.45 (0.09)

Abbreviations: RA, rheumatoid arthritis; ACPA, anti-citrullinated protein antibody; SD, standard deviation; DAS28, disease activity score in 28 joints; VAS, visual analogue scale; pSTAT, phosphorylated signal transducer and activator of transcription; RFU, relative fluorescence units.

After blood sampling, 18 patients with recent-onset RA started DMARD therapy according to the national guidelines [35] and EULAR recommendations [2]: 8 patients (42%) methotrexate (MTX)-based combination, 3 patients (16%) other combination, 5 patients (26%) MTX monotherapy, and 2 patients (11%) other monotherapy. In addition, 6 patients (32%) started a course of low-dose (≤ 10 mg/day) oral prednison. During follow-up the drug treatment was modified, targeting to remission, in line with the national and EULAR recommendations [35,2]. At follow-up, 1 patient had stopped DMARD treatment and 3 patients had stopped oral prednison. The EULAR treatment response was good in 11 patients (58%), moderate in 2 patients (11%), and 6 patients (32%) did not respond.

In the chronic RA group, 14 patients started an anti-TNF drug (5 golimumab, 4 etanercept, 3 adalimumab, and 2 certolizumab), 1 patient started tocilizumab, and 1 patient rituximab. In addition to biologicals, 6 patients (38%) used a MTX-based DMARD combination, 4 patients (25%) other DMARD combination, 2 patients (13%) MTX monotherapy, and 2 patients (13%) other DMARD monotherapy. In addition, 9 patients (56%) used low-dose (≤ 10 mg/day) oral prednison. During follow-up one patient stopped rituximab treatment and 3 patients were switched to another TNF blocker. The EULAR treatment response was good in 10 patients (63%), moderate in 3 patients (19%), and 3 patients (19%) did not respond.

At baseline, pSTAT6 and pSTAT1 levels and the pSTAT1/pSTAT6 ratio did not correlate with age, erythrocyte sedimentation rate, or CRP (Table 2).

**Table 2:** Correlation of signaling results with age and inflammatory markers at baseline in all patients.

	Age, years	ESR, mm/h	CRP, mg/l
Baseline	r (95% CI)	r (95% CI)	r (95% CI)
*Monocyte*			
pSTAT6, RFU	0.14 (-0.21 to 0.45)	0.03 (-0.30 to 0.36)	0.11 (-0.23 to 0.43)
pSTAT1, RFU	-0.20 (-0.50 to 0.14)	0.08 (-0.26 to 0.40)	0.28 (-0.06 to 0.56)
pSTAT1/pSTAT6 ratio	-0.32 (-0.59 to 0.01)	-0.01 (-0.34 to 0.33)	0.18 (-0.16 to 0.49)
*Lymphocyte*			
pSTAT6, RFU	0.20 (-0.14 to 0.50)	0.12 (-0.22 to 0.44)	0.17 (-0.17 to 0.48)
pSTAT1, RFU	-0.06 (-0.38 to 0.28)	0.26 (-0.08 to 0.55)	0.26 (-0.08 to 0.54)
pSTAT1/pSTAT6 ratio	-0.25 (-0.54 to 0.10)	0.02 (-0.31 to 0.36)	0.01 (-0.32 to 0.34)

ESR, erythrocyte sedimentation rate; CRP, C-reactive protein; r, Spearman correlation coefficient; CI, confidence interval; pSTAT, phosphorylated signal transducer and activator of transcription, RFU, relative fluorescence units.

### pSTAT6 predicts treatment response to DMARDs in recent-onset RA and decreases during successful treatment

Among patients with recent-onset RA, baseline STAT6 phosphorylation (pSTAT6) levels upon IL-4 stimulation in monocytes were higher in those who achieved good response to DMARDs than in those who did not (Table 3). IFN-γ -stimulated pSTAT1 levels and the ratio of IFN-γ-stimulated pSTAT1 to IL-4 -stimulated pSTAT6 of these patients at baseline were not associated with response to DMARDs (Table 3). Also, pSTAT6 and pSTAT1 levels and the pSTAT1/pSTAT6 ratio did not correlate with disease activity determined by DAS28 (data not shown).

**Table 3:** Univariate odds ratios of good EULAR response calculated by estimation-based exact logistic regression.

	Recent-onset RA		Chronic RA	
Baseline	OR* (95% CI)	p value	OR* (95% CI)	p value
*Monocyte*				
pSTAT6, RFU	2.74 (1.05 to 9.47)	0.037	1.91 (0.64 to 6.72)	0.27
pSTAT1, RFU	2.45 (0.76 to 9.66)	0.14	1.68 (0.48 to 6.72)	0.44
pSTAT1/pSTAT6 ratio	0.86 (0.25 to 2.85)	0.81	1.08 (0.36 to 3.35)	0.89
*Lymphocyte*				
pSTAT6, RFU	2.25 (0.91 to 7.40)	0.084	2.37 (0.81 to 11.25)	0.13
pSTAT1, RFU	1.76 (0.63 to 5.58)	0.30	3.91 (1.12 to 20.68)	0.029
pSTAT1/pSTAT6 ratio	0.59 (0.22 to 1.41)	0.25	0.85 (0.33 to 2.09)	0.74

* per 1 standard deviation.

Abbreviations: EULAR, European League Against Rheumatism; RA, rheumatoid arthritis; OR, odds ratio; CI, confidence interval; pSTAT, phosphorylated signal transducer and activator of transcription, RFU, relative fluorescence units.

**Fig 2:**
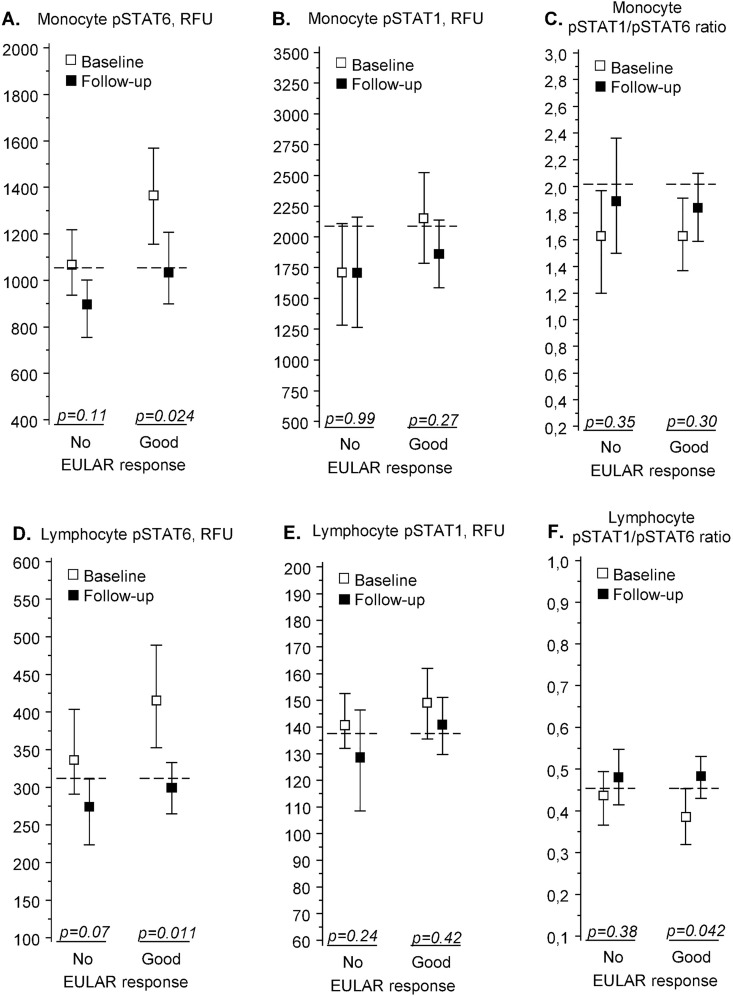
pSTAT6 and pSTAT1 in patients with recent-onset rheumatoid arthritis (n = 19). IL-4-induced pSTAT6 (A, D) and IFN-γ-induced pSTAT1 (B, E) fluorescence intensities and their ratios (C, F) in peripheral blood monocytes (A-C) and lymphocytes (D-F). Measurements were made before treatment (“Baseline”) and after 1-year treatment with disease-modifying antirheumatic drugs (“Follow-up”). Results are shown separately for patients with no or moderate treatment response (“No”, n = 7) or good response (“Good”, n = 12), as defined by EULAR criteria. The fluorescence intensities are given in relative fluorescence units (RFU), with squares denoting group means and whiskers denoting 95% confidence intervals. The horizontal dashed lines show the corresponding mean values in healthy controls (n = 37). The p values denote significance of difference between baseline and follow-up values (permutation test). Predictive value of baseline levels for treatment response are shown in Table 2. Abbreviations: pSTAT, phosphorylated signal transducer and activator of transcription, EULAR, European League Against Rheumatism.

During follow-up, pSTAT6 levels fell significantly among good responders in monocytes and lymphocytes (Fig 2A and 2D), while there were no significant changes in pSTAT1 levels (Fig 2B and 2E). This led to an increase in the pSTAT1/pSTAT6 ratios (Fig 2C and 2F), which was significant in good responders’ lymphocytes (Fig 2F).

### pSTAT1 predicts treatment response to biological drugs in chronic RA

Among patients with chronic RA, baseline pSTAT1 levels upon IFN-γ stimulation in lymphocytes were higher in those who achieved good response to biological drug in than in those who did not (Table 3). IL-4 -stimulated pSTAT6 levels and the ratio of IFN-γ-stimulated pSTAT1 to IL-4 -stimulated pSTAT6 of these patients at baseline were not associated with response to biological treatment (Table 3). pSTAT6 and pSTAT1 levels and the pSTAT1/pSTAT6 ratio did not correlate with disease activity determined by DAS28 (data not shown).

**Fig 3:**
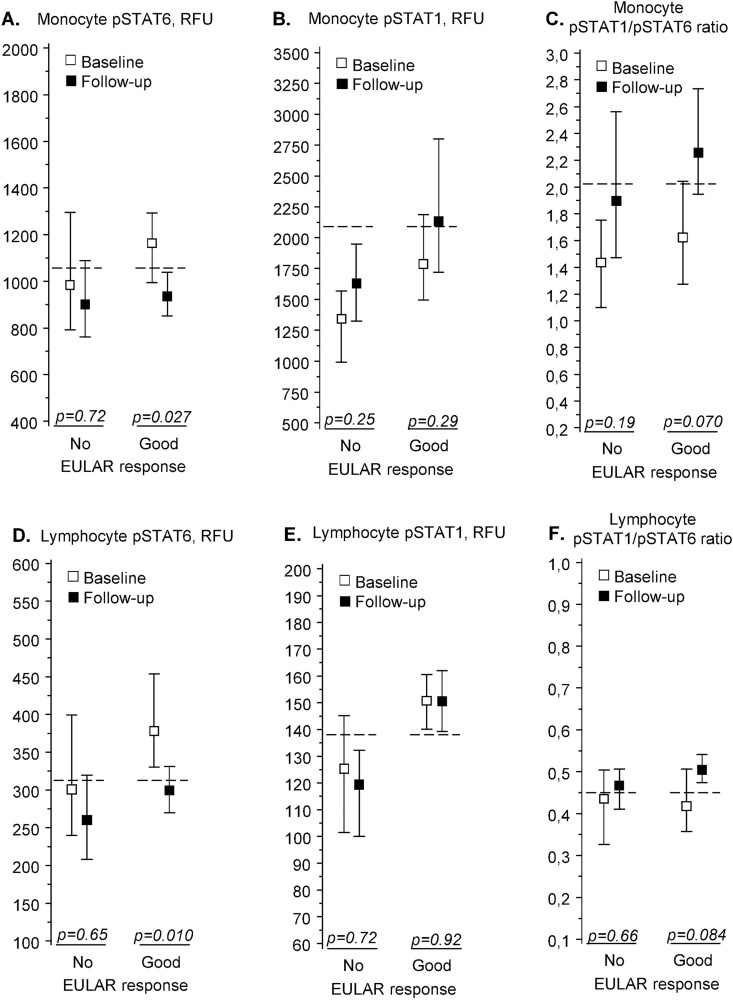
pSTAT6 and pSTAT1 in patients with chronic rheumatoid arthritis (n = 16). IL-4 -induced pSTAT6 (A, D) and IFN-γ -induced pSTAT1 (B, E) fluorescence intensities and their ratios (C, F) in peripheral blood monocytes (A-C) and lymphocytes (D-F). Measurements were made before treatment (“Baseline”) and after 1-year treatment with biological drugs (“Follow-up”). Results are shown separately for patients with no or moderate treatment response (“No”, n = 6) or good response (“Good”, n = 10), as defined by EULAR criteria. The fluorescence intensities are given in relative fluorescence units (RFU), with squares denoting group means and whiskers denoting 95% confidence intervals. The horizontal dashed lines show the corresponding mean values in healthy controls (n = 37). The p-values denote significance of difference between baseline and follow-up values (permutation test). Predictive value of baseline levels for treatment response are shown in Table 2. Abbreviations: pSTAT, phosphorylated signal transducer and activator of transcription, EULAR, European League Against Rheumatism.

During follow-up, pSTAT6 levels fell significantly in monocytes and lymphocytes (Fig 3A and 3D), while there were no significant changes in pSTAT1 levels (Fig 3B and 3E). This led to a non-significant but clear increase in the pSTAT1/pSTAT6 ratios in good responders’ monocytes and lymphocytes (Fig 3C and 3F).

## Discussion

Our results reveal that baseline STAT6 phosphorylation level, as determined by whole blood phospho-specific flow cytometry, in monocytes upon IL-4 stimulation is positively correlated with good treatment response to synthetic DMARDs in patients with recent-onset untreated RA, and, that baseline STAT1 phosphorylation level in lymphocytes upon IFN-γ stimulation is positively correlated with good treatment response to biological drugs in patients with chronic RA. The findings are novel and suggest that activation capability of STAT6 and STAT1 signaling pathways of circulating leukocytes may aid to predict treatment response in RA patients. We also found recently that baseline STAT3 phosphorylation in peripheral blood CD4^+^ T cells is associated with good treatment response to synthetic DMARDs in recent-onset RA [36]. Immune cell profiling strategies other than above have also been applied to find predictive markers in RA [37]. These include determination of the expression of chemokine receptors on T cells and monocytes for predicting response to infliximab [17], CD28 on T cells for predicting response to abatacept [18], CD16 on monocytes for predicting response to methotrexate [19], and ZAP-70 in B cells for predicting response to rituximab [38]. Taken together, immune cell profiling is a promising approach to be used in guiding personalized treatment decisions and improving outcomes of patients with RA.

The mechanisms underlying the positive correlation between STAT6 phosphorylation level and response to DMARDs are not known but may involve priming of circulating cells. Indeed, inflammatory stimuli like autoantibodies and Toll-like receptor ligands may up-regulate IL-4 receptor expression to cause priming for STAT6 signaling in myeloid cells. This, in turn, can limit excessive inflammation and tissue damage. [39] Also, high percentage of IL-4 positive CD4^+^ T cells at onset of RA has been reported to predict remission with methotrexate treatment [40]. Furthermore, it has been shown in mouse models that deficiency of IL-4 and STAT6 can result in significant increase in arthritis severity [28], and that overexpression of IL-4 may protect from cartilage erosions [41]. Hence, good response to DMARDs in recent-onset RA may be associated with monocytes’ good capability for IL-4 -mediated responses that protect from progression of tissue destruction associated with RA.

Our observation that there is a positive correlation between IFN-γ -stimulated STAT1 phosphorylation level in circulating lymphocytes and treatment response to biological drugs in patients with chronic RA may reflect an overall immunological state that is facilitated by good STAT1 activation capability. Supporting this concept, it has been reported recently that IFN-γ inhibits IL-17 production in a STAT1-dependent manner, thus representing one mechanism favoring the development of Th1 cells and silencing the Th17 program [42]. Furthermore, Ortiz *et al*. showed that levels of IFN-γ -induced pSTAT1 positive cells in peripheral blood of RA patients correlate inversely with the number of memory T cells, and, close to our finding, that baseline levels of the IFN-γ -induced pSTAT1+ cells are higher in those RA patients who obtain good response to the IL-6 blocker tocilizumab [43]. Altogether, it is possible that higher capacity to phosphorylate STAT1 in response to IFN-γ can be a marker able to distinguish the RA patients who are amenable to treatment with TNF or IL-6 blockers. This needs to be verified in larger studies, as well as the possibility that the same applies to additional biologicals.

In the present study we also found that STAT6 phosphorylation decreases during treatment, which agrees with the finding that STAT6 expression decreases in response to successful DMARD treatment in the RA synovium [21]. The mechanisms that are able to attenuate STAT6 phosphorylation include negative feedback provided by members of the suppressors of cytokine signaling family, which may regulate IL-4-dependent STAT6 activation [44] and are elevated in RA patients’ peripheral blood mononuclear cells [45]. As to lymphocyte STAT1 phosphorylation in chronic RA patients not responding to multiple synthetic DMARDs, high baseline levels predicted good response to biologicals and did not decrease during the therapy. If confirmed in larger studies, STAT1 phosphorylation could serve as a predictive surrogate marker identifying a subgroup of patients with chronic RA who will have good treatment response to biologicals.

In order to evaluate the relationship of combined markers with the treatment response, we calculated the ratio of IFN-γ -stimulated STAT1 phosphorylation to IL-4 -stimulated STAT6 phosphorylation in leukocytes. The ratio may indicate how the immune response types are orientated, especially as IFN-γ has been shown sufficient for Th1 differentiation, whereas IL-4 is critical for Th2 differentiation [46]. We found no association between the pSTAT1/pSTAT6 ratio at baseline and treatment response, but, however, the ratio increased during treatment with synthetic or biological DMARDs, and the increase was significant in lymphocytes during successful DMARD treatment in recent-onset RA. Our findings are consistent with results showing that a shift to the Th1 direction occurred in the Th1/Th2 cell ratio in patients with RA during either TNF blocker or glucocorticoid treatment [47], and that the expression of IFN-γ compared to that of IL-4 in peripheral blood mononuclear culture increased under treatment with the TNF antibody infliximab [48]. IFN-γ can suppress IL-4 -induced STAT6 activation, as observed in monocytes and Th1 cells [49,50]. Altogether, the increased pSTAT1/pSTAT6 ratio after follow-up observed in our study seems to be more due to a decrease in STAT6 phosphorylation capability than an increase in STAT1 phosphorylation capability.

Although pSTAT1 and pSTAT6 predicted treatment response, of interest, they did not correlate with DAS28, suggesting that the two markers are distinct from disease activity markers. The only correlation observed between disease activity measures and pSTAT6 and pSTAT1 phosphorylation was a positive correlation between the pSTAT1/pSTAT6 ratio at study entry and swollen joint count. This feature may reflect the potent effects of the IFN-γ/STAT1 pathway within joints, including priming of monocytes and macrophages and induction of inflammatory mediator production [51]. This is likely to represent the complexity of the cellular and molecular interactions involving IFN-γ/STAT1 and IL-4/STAT6 pathways during different phases of RA, which is also evident in the opposing results obtained from other studies. There are studies showing that in human peripheral blood leukocytes, the IFN-γ/STAT1 pathway is able to limit cellular infiltration and potential tissue damage at inflammatory sites [52,53]. Also, in certain mouse models, the IFN-γ/STAT1 pathway mediates protective effects in autoimmune disease and arthritis [24], and lack of IL-4 and STAT6 suppresses arthritis [27]. However, in other mouse models, the IFN-γ/STAT1 pathway has increased and the IL-4/STAT6 pathway decreased the severity of arthritis [28]. Clearly, further studies are required to reveal the mechanisms explaining our results on the associations of IL-4/STAT6 and IFN-γ/STAT1 signaling in immune cells and the course of RA.

A lthough there was significant association between STAT6 and STAT1 phosphorylation levels and treatment response in RA, the levels overlapped with those of healthy reference subjects. However, among the numerous inflammatory pathways operating in RA, it is important to find the specific molecular markers that are able to distinguish the patients who will respond to a given treatment from those who will not, even if the markers serve as surrogate markers. As the method used in the present study allows determining phosphorylation and/or expression of several targets simultaneously, it is applicable for combining or correlating STAT1 and STAT6 phosphorylation with other markers, thereby possibly creating markers that can be utilized in tailoring personalized treatment for patients with RA in the future. The patients studied were well characterized with rigorous inclusion criteria, although the number of patients brands this a pilot study.

## Conclusion

Our current results show that baseline STAT6 and STAT1 phosphorylation levels in circulating leukocytes are associated with treatment response to synthetic DMARDs and biologicals in RA, and that their ratio is influenced by the treatments. If confirmed in larger studies, the results may be utilized in developing personalized medicine for patients with RA.
